# High Expression of Nicotinamide *N*-Methyltransferase in Patients with Sporadic Alzheimer’s Disease

**DOI:** 10.1007/s12035-020-02259-9

**Published:** 2021-01-02

**Authors:** Altin Kocinaj, Tabassum Chaudhury, Mohammed S. Uddin, Rashad R. Junaid, David B. Ramsden, Geshanthi Hondhamuni, Fábio Klamt, Linda Parsons, Richard B. Parsons

**Affiliations:** 1 Institute of Pharmaceutical Science, King’s College London, 150 Stamford Street, London, SE1 9NH UK; 2 Institute of Metabolism and Systems Research, University of Birmingham, Edgbaston, Birmingham, B15 2TH UK; 3 Queen Square Brain Bank for Neurological Disorders, UCL Queen Square Institute of Neurology, University College London, 1 Wakefield Street, London, WC1N 1PJ UK; 4 Laboratory of Cellular Biochemistry, Universidade Federal do Rio Grande do Sul, 2600 Ramiro Barcelos St., Porto Alegre, RS 90035-003 Brazil; 5 National Institute of Science and Technology – Translational Medicine (INCT-TM), Porto Alegre, Brazil

**Keywords:** Alzheimer’s disease, Nicotinamide *N*-methyltransferase, Pathogenic process, Overexpression, Therapies, Homocysteine, Stress response

## Abstract

We have previously shown that the expression of nicotinamide *N*-methyltransferase (NNMT) is significantly increased in the brains of patients who have died of Parkinson’s disease (PD). In this study, we have compared the expression of NNMT in post-mortem medial temporal lobe, hippocampus and cerebellum of 10 Alzheimer’s disease (AD) and 9 non-disease control subjects using a combination of quantitative Western blotting, immunohistochemistry and dual-label confocal microscopy coupled with quantitative analysis of colocalisation. NNMT was detected as a single protein of 29 kDa in both AD and non-disease control brains, which was significantly increased in AD medial temporal lobe compared to non-disease controls (7.5-fold, *P* < 0.026). There was no significant difference in expression in the cerebellum (*P* = 0.91). NNMT expression in AD medial temporal lobe and hippocampus was present in cholinergic neurones with no glial localisation. Cell-type expression was identical in both non-disease control and AD tissues. These results are the first to show, in a proof-of-concept study using a small patient cohort, that NNMT protein expression is increased in the AD brain and is present in neurones which degenerate in AD. These results suggest that the elevation of NNMT may be a common feature of many neurodegenerative diseases. Confirmation of this overexpression using a larger AD patient cohort will drive the future development of NNMT-targetting therapeutics which may slow or stop the disease pathogenesis, in contrast to current therapies which solely address AD symptoms.

**Supplementary Information:**

The online version contains supplementary material available at 10.1007/s12035-020-02259-9.

## Background

Alzheimer’s disease (AD) is the most common form of dementia [1]. AD is a synaptopathy, characterised by the loss of synapses which predates the onset of symptoms by several years [2, 3]. The mechanisms underlying synaptopathy remain unclear; however, both the accumulation and aggregation of the amyloidogenic protein amyloid-β (Aβ), in particular Aβ_1–42_, and the hyperphosphorylation and subsequent aggregation of the cytoskeletal protein tau have been linked to the loss of synapses observed in AD [4, 5]. Currently, there is no cure for the pathogenic process, with treatments being palliative and symptomatic [6].

Nicotinamide *N*-methyltransferase (NNMT, E.C. 2.1.1.1) is responsible for the *N*-methylation of nicotinamide to 1-methylnicotinamide (MeN) [7]. In the human brain, NNMT is a solely neuronal protein with varied levels of expression in regions of the brain [8]. NNMT expression is significantly increased in the brains of patients who have died of Parkinson’s disease (PD) [8, 9]. Our subsequent in vitro studies demonstrated that ectopic expression of NNMT in the SH-SY5Y human neuroblastoma cell-line had multiple cytotrophic effects, including increased complex I activity and ATP synthesis, and protection against a range of PD-relevant mitotoxins such as rotenone, 1-methyl-4-phenylpyridinium ion and 6-hydroxydopamine [10, 11]. Increased complex I activity was due to the induction and activation of sirtuins 1 and 3, DNA histone deacetylases linked with longevity and cell survival [12]. Moreover, NNMT expression increased the formation of functional synapses, mediated via the activation of the ephrin-B2/Akt signalling pathway [13]. Hence, we proposed that the increase in NNMT expression observed in PD patients may be a stress response of the neurone to the disease pathogenesis [10, 12, 13].

It is possible that the high expression of NNMT we observe in PD is not unique and may be a feature of other neurodegenerative diseases. To investigate this, we have undertaken a small-cohort proof-of-principle study to compare the expression levels of NNMT protein in post-mortem medial temporal lobe (MTL) and hippocampus, areas of significant AD-related pathology, of AD and non-disease control (NDC) subjects. In addition, we investigated the cell-type expression of NNMT in these same tissues. NNMT protein levels and cell-type expression were also investigated in the cerebellum.

## Methods

Unless otherwise stated, all reagents were obtained from Sigma and were of the highest purity available.

### Human Tissue

Post-mortem tissue from 10 AD (age range 61–88) and 9 NDC (53–89) was obtained from the Queen Square Brain Bank for Neurological Disorders, University College London. The average ages were not significantly different between NDC and AD subjects (82.4 ± 11.3 and 72.6 ± 8.4 years for NDC and AD subjects, respectively, *P* = 0.0503 using Student’s *t* test with Welch correction). Where precise data were available, post-mortem intervals were found not to be significantly different between NDC and AD subjects (46.4 ± 26.5 vs. 53.25 ± 15.5 h, *P* = 0.62 using Student’s *t* test with Welch correction, *n* = 5 and *n* = 8 for NDC and AD subjects, respectively). The NDC group of subjects comprised 6 females and 3 males and the AD group of subjects comprised 6 females and 4 males, proportions which match the 2:1 female:male AD prevalence ratio in the population [14, 15]. AD subjects comprised solely sporadic cases with no known family history of disease. NDC subjects comprised cases who were all cognitively normal with no overt symptoms of AD. Subjects with a history of cancer were excluded from both cohorts due to the increased expression of NNMT observed in this disease [8, 9, 16, 17]. Available pathological details of NDC and AD subjects are summarised in Table 1.

**Table 1:** Subject data. Although the majority of non-disease control patients demonstrated low levels of Alzheimer’s-related pathology, clinically they did not present with symptoms, a requirement for the diagnosis of Alzheimer’s disease

Subject #	Age	Sex	PMI^a^	Braak stage	CERAD	Neuropathological comorbidity
Non-disease controls						
NDC1	87	F	53	I	Sparse	None
NDC2	87	F	21	II	-^b^	Mild vascular CAA^c^, cerebral infarct
NDC3	87	M	> 24	II	-	-
NDC4	88	M	17	II	Sparse	None
NDC5	82	F	> 24	II	-	-
NDC6	89	F	64	II	-	Mild vascular CAA, cerebral infarct
NDC7	87	M	> 24	I	-	-
NDC8	82	F	77	II	-	Mild vascular CAA
NDC9	53	F	> 24	0	-	-
Alzheimer’s disease						
AD1	67	F	36	VI	-	Severe vascular CAA
AD2	80	F	32	VI	-	-
AD3	73	M	45	VI	High	None
AD4	61	M	> 24	VI	-	-
AD5	69	M	50	VI	High	None
AD6	62	F	77	VI	-	None
AD7	88	M	58	VI	Moderate	Severe vascular CAA, TDP43opathy
AD8	76	F	60	VI	High	Moderate vascular CAA, TDP43opathy
AD9	79	F	68	VI	-	None
AD10	71	F	> 24	VI	-	-

^a^Post-mortem interval, expressed in hours

^b^Not recorded

^c^Cerebral amyloid angiopathy

### Quantitative Western Blotting

Flash-frozen MTL and cerebellum tissues (500 mg) were prepared for SDS-PAGE/Western blotting as per previously described [8] using radioimmunoprecipitation assay buffer comprising 50 mM Tris-HCl pH 7.5, 150 mM NaCl, 1% v/v nonidet P-40, 5 mM ethylenediaminetetraacetic acid, 0.5% w/v sodium deoxycholate and 0.1% w/v sodium dodecylsulphate. Samples were analysed using Western blotting three times, with NNMT and the loading control protein β-tubulin detected using combinations of primary and secondary antibodies outlined in Table 1. Bands were visualised using electrochemiluminescence detection and quantified using densitometry. The NNMT protein level for each replicate was calculated as normalised tubulin ratio, and NNMT expression for each subject was calculated as the mean of the three independent replicates. NNMT protein expression for each group was calculated as the average of all subjects and expressed as normalised tubulin ratios ± SEM.

### 3.3′-Diaminobenzidine Immunohistochemistry

Formalin-fixed, paraffin-embedded sections (7 μm) of MTL and cerebellum were prepared on glass slides and subjected to immunohistochemistry as previously described [8, 9]. Briefly, sections were deparaffinised in multiple changes of xylene, demyelinated using chloroform, and endogenous peroxidase activity was exhausted using methanolic H_2_O_2_ (0.3% v/v). Sections were rehydrated through descending grades of alcohol and permeabilised using 0.2% (v/v) nonidet-P40. Proteins were detected using primary antibodies as outlined in Table 2, with secondary antibody detection using the VectaStain ABC detection kit (Vector Laboratories, Peterborough, UK). Sections were stained using 3,3′-diaminobenzidine (DAB) and counterstained using Mayer’s haematoxylin. Sections were dehydrated and mounted in DPX mounting medium (Fisher Scientific, Loughborough, UK). Sections were imaged using a Leica microscope (Leica Microsystems, Milton Keynes, UK).

**Table 2:** Primary and secondary antibodies used for Western blotting, immunohistochemistry and confocal microscopy

Protein	Primary antibody			Secondary antibody	
	Western blotting	IHC/Confocal microscopy^a^	Product code	Western blotting	Confocal microscopy
Nicotinamide *N*-methyltransferase	2.5 μg/mL	1:50	ab58743 (Abcam)	1:2000 (A8275, Sigma)	AlexaFluor™ 488 (A32731, Molecular Probes)
Choline acetyltransferase	-	1:50 (IHC)	GTX113163 (GeneTex)	-	-
		1:100 (Confocal)	ab34419 (Abcam)		AlexaFluor™ 594 (A32759, Molecular Probes)
Glial acidic fibrillary protein	-	1:100	ab7260 (Abcam)	-	AlexaFluor™ 594 (A32744, Molecular Probes)
Tubulin	1:1000	-	ab180207 (Abcam)	1:5000 (A4416, Sigma)	-

*IHC* 3,3′-diaminobenzidine immunohistochemistry

^a^Unless otherwise stated, the quoted antibody dilution was used for both 3,3′-diaminobenzidine immunohistochemistry and dual-label confocal microscopy

### Dual-Label Confocal Microscopy

Formalin-fixed, paraffin-embedded hippocampus sections (7 μm) were prepared, dewaxed and rehydrated as described above. Cellular NNMT expression was determined using a combination of NNMT and various cell-type markers as outlined in Table 2, with secondary antibody detection using AlexaFluor 488 and AlexaFluor 594-conjugated secondary antibodies for NNMT and cellular markers, respectively. Sections were counterstained using DAPI and mounted in ProLong Gold™ antifade mountant (Life Technologies, UK). Images were captured using a Nikon A1 inverted confocal microscope using a × 20 objective lens as z-stacks at a resolution of 1024 × 1024 pixels per inch, which were rendered into maximum projection images.

Confocal microscopy images were quantified using the EzColocalization plugin v1.1.3 in FIJI ImageJ v1.53a [18]. Maximum projection images were rendered into single-channel .tiff image files which were used as inputs for analysis. Colocalisation was assessed using linear threshold overlap score (TOS) [19] with thresholds calculated using the method of Costes [20] and expressed as TOS ± SD. Additionally, channel overlap (i.e. overlap between NNMT and each cell marker) was calculated using Mander’s colocalisation coefficients [21] using Costes’ thresholds and expressed as M1 and M2 ± SD.

### Statistical Analysis

All statistical comparisons were performed using Prism v8.0 (GraphPad, San Diego, USA). For Western blotting results, statistical comparison of NNMT expression between (i) NDC vs. AD MTL and cerebellum and (ii) AD subjects with and without neuropathological comorbidities was performed using two-tailed unpaired Student’s *t* test with Welch correction. For colocalisation experiments, three statistical analyses were conducted upon TOS, M1 and M2 values using multiple *t* tests with correction for multiple comparisons using the Holm-Sidak method: (i) comparison of ChAT values in NDC and AD subjects, (ii) comparison of GFAP values in NDC and AD subjects and (iii) comparison of ChAT and GFAP values in pooled subjects. *P* < 0.05 was used to indicate statistical significance.

## Results

### NNMT Is Overexpressed in AD Medial Temporal Lobe

NNMT protein expression was compared in the MTL of AD and NDC subjects using quantitative Western blotting. MTL was used instead of hippocampus because of the extensive hippocampal neurodegeneration observed in these patients and the involvement of the MTL in disease progression [22]. Cerebellum was chosen because it is a brain region which, although it is affected in AD, has limited microglial pathology, no reduction in Purkinje and granule neurone number and a reduction in volume which is lower than that observed in AD cerebral cortices. Hence, its involvement is considered to be secondary to that of the cerebral cortices [23–25].

**Fig. 1:**
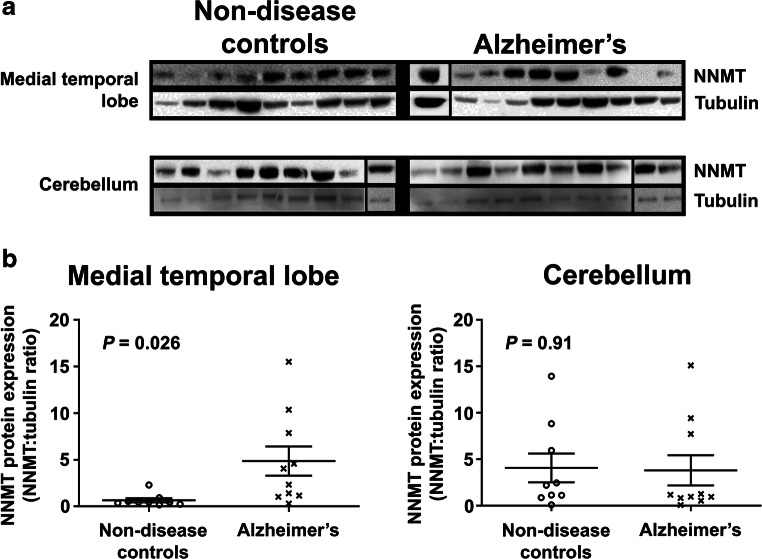
Nicotinamide *N*-methyltransferase protein expression is significantly increased in Alzheimer medial temporal lobe. **a** Representative Western blots of RIPA-soluble lysates prepared using medial temporal lobe (MTL) and cerebellum from non-disease control (NDC) and Alzheimer’s (AD) subjects. Samples were probed for nicotinamide *N*-methyltransferase (NNMT) and β-tubulin (tubulin) using anti-NNMT and anti-tubulin antibodies and bands visualised using electrochemiluminescence detection. Due to the limitations of the sizes of the gel, samples were loaded over more than one gel; however, NNMT and tubulin for each sample were detected using the same gel. In each group, samples electrophoresed on different gels are shown separated by a black vertical line. NNMT and tubulin for each replicate sample were detected and quantified using the same gel, and all samples in each replicate were processed in parallel. **b** Quantification of NNMT expression. Bands were quantified using densitometry using ImageJ; after which, NNMT was normalised for tubulin expression and expressed as normalised tubulin ratios ± SEM. Statistical analysis was performed using two-way unpaired Student’s *t* test, *n* = 9 for NDC subjects and *n* = 10 for AD subjects, *P* = 0.026 for MTL and *P* = 0.91 for cerebellum

NNMT was observed as a single protein of approximately 29 kDa in the MTL and cerebellum of both NDC and AD subjects (Fig. 1a), with protein band intensity varying between subjects. Densitometric quantitation revealed that NNMT protein expression was significantly increased in AD MTL compared to NDC (Fig. 1b, 4.86 ± 1.57 vs. 0.65 ± 0.2, *n* = 10 and 9, respectively, *P* = 0.026). There was no significant difference in cerebellar NNMT protein expression for AD and NDC subjects (Fig. 1b, 3.81 ± 1.62 vs. 4.07 ± 1.55, *n* = 10 and 9, respectively, *P* = 0.91).

For AD subjects where measures of pathology severity were available, all were of Braak stage VI, of high CERAD scores and they exhibited significant vascular pathology (Table 1); hence, it was not possible to stratify these subjects by severity of pathology. There was no difference in NNMT expression between AD subjects with and without neuropathological comorbidities (5.32 ± 2.72 vs. 6.27 ± 7.0, *P* = 0.84, *n* = 3 and 4, respectively).

There was no significant difference in NNMT protein expression between male and female subjects in either the MTL or the CBM, either as a complete cohort or when stratified into NDC and AD subjects (Table S1). There was no correlation between NNMT protein expression in the MTL and cerebellum, either as a complete cohort or when stratified by disease status (Table S2). Likewise, there was no correlation between NNMT protein expression and age at death (Table S3) or post-mortem interval (Table S4).

### NNMT Is Expressed Solely in Neurones of the Medial Temporal Lobe

**Fig. 2:**
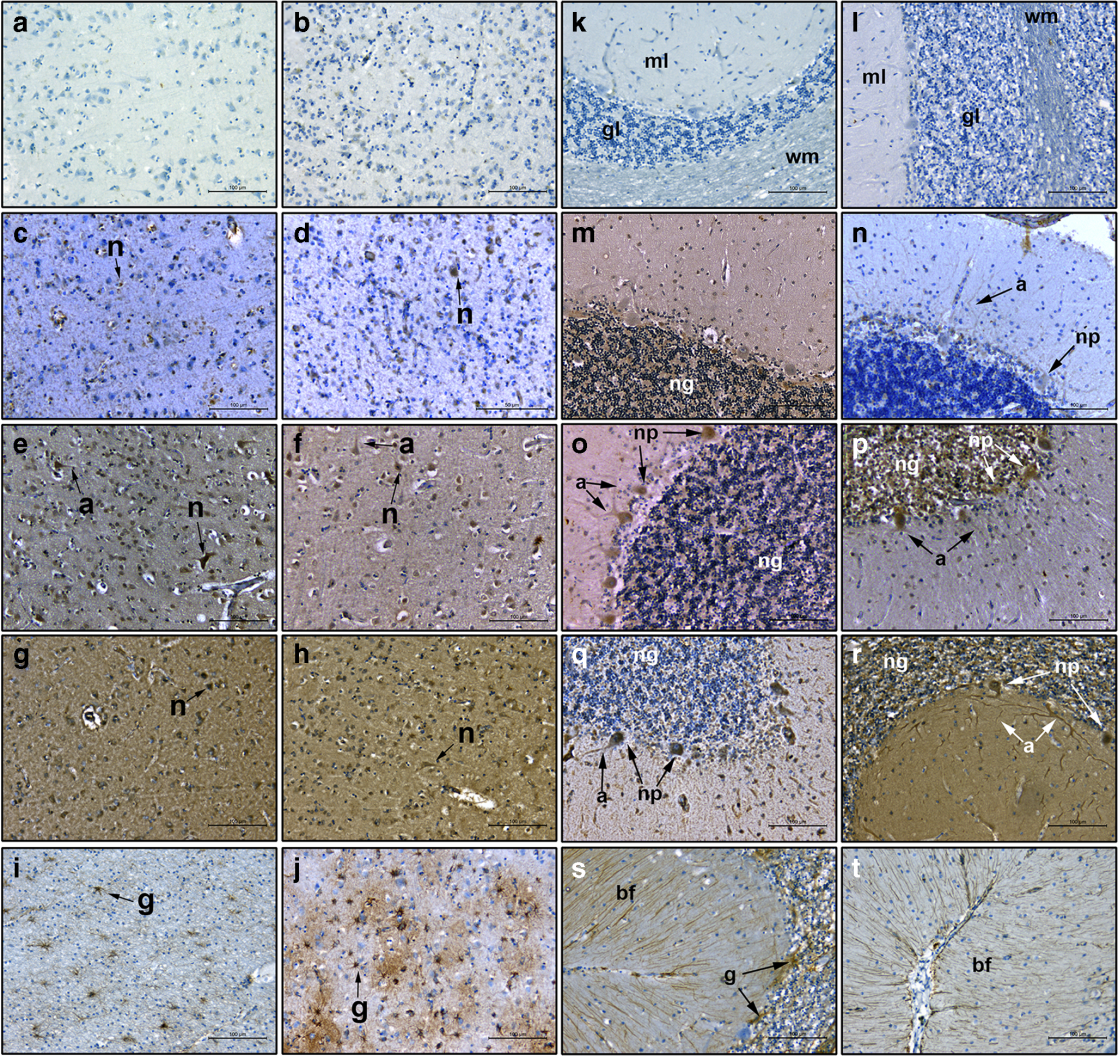
NNMT expression in the medial temporal lobe and cerebellum. The expression of NNMT, the cholinergic neurone marker choline acetyltransferase (ChAT) and the glial marker glial acidic fibrillary protein (GFAP) were detected using antibodies against each and visualised using 3,3′-diaminobenzidine immunohistochemistry in MTL (**a**–**j**) and cerebellum (**k**–**t**). Counterstaining was performed using haematoxylin. **a** and **b** Staining of NDC and AD tissue in the absence of primary antibody. **c** and **d** Anti-NNMT staining in NDC and AD subjects with low NNMT expression respectively. **e** and **f** Anti-NNMT staining in NDC and AD subjects with high NNMT expression, respectively. **g** and **h** Anti-ChAT staining in NDC and AD subjects, respectively. **i** and **j** Anti-GFAP staining in NDC and AD subjects, respectively. **k** and **l** Staining of NDC and AD tissue in the absence of primary antibody. **m** and **n** Anti-NNMT staining in NDC and AD subjects with low expression, respectively. **o** and **p** Anti-NNMT staining in NDC and AD subjects with high NNM expression, respectively. **q** and **r** Anti-ChAT staining in NDC and AD subjects, respectively. **s** and **t** Anti-GFAP staining in NDC and AD subjects, respectively. For all panels, n, neurone; a, axon; g, glial cell; ml, molecular layer; gl, granule layer; wm, white matter; ng, granule layer neurones; np, Purkinje neurones; bf, Bergmann fibres. For each, scale bar = 100 μm

Having shown that NNMT was significantly overexpressed in the MTL of AD subjects, we next determined whether NNMT expression was limited to neurones in disease-affected areas, as previously reported in PD patient brain [8]. NNMT expression was examined in all subjects and examples typical of staining are shown in Fig. 2. No staining was evident in either NDC or AD MTL when primary antibodies were replaced with antibody diluent only (panels a and b). NNMT expression in both NDC and AD MTL was solely neuronal (panels c–f). In NDC and AD subjects with low NNMT expression (panels c and d), staining was limited to the cell body, with little axonal staining evident. In NDC and AD subjects with high NNMT expression (panels e and f), cell body staining was much more intense, with evidence of extensive axonal and neurite staining. Staining of both NDC and AD MTL with anti-choline acetyltransferase (ChAT), antibody showed solely neuronal expression, with morphology identical to those neurones expressing NNMT (panels g and h). Glial acidic fibrillary protein (GFAP) staining was present throughout the MTL in both NDC and AD subjects, with GFAP-positive cells presenting a stellate morphology representative of activated glia which was different to the morphology of cells expressing NNMT (panels i and j). AD subjects demonstrated evidence of extensive gliosis as shown by an increase in both the number and intensity of GFAP-positive cells (panel i), plus an increase in their stellate morphology (panel j), compared to NDC.

Having shown that neurones of the MTL express NNMT and that these are likely to be cholinergic neurones, the next step was to determine whether NNMT was expressed in cholinergic neurones in other brain regions or whether it was limited solely to the MTL. For this, the cerebellum was chosen due to its lack of AD pathology. As with MTL, no staining was evident when primary antibodies were replaced with antibody diluent only (panels k and l). NNMT expression in cerebellum was also solely neuronal in both AD and NDC subjects (panels m–p), with no glial localisation present. In NDC subjects with low NNMT expression (panel m), staining was limited to the granule neurones, with no Purkinje cell staining evident. In NDC subjects with high NNMT expression (panel o), NNMT staining was also observed in the Purkinje neurones and their axons within the molecular layer. In AD subjects, staining was evident in both the granule and the Purkinje neurones and their associated axons in both low and high-expressing subjects (panels n and p). Staining with anti-ChAT antibody suggested that these neurones were cholinergic in phenotype (panels q and r). Intense ChAT staining was observed in the cell bodies of Purkinje cells and their axons, along with expression in some granule neurones, in NDC subjects (panel q). In AD subjects, ChAT staining was observed in a significant number of granule neurones (panel r). Furthermore, ChAT staining was extensive within the axons and the dendritic tree of the Purkinje neurones. Finally, GFAP staining was intense throughout the granule and molecular layers of the cerebellum, with extensive Bergman fibres observed in the molecular layer, of both NDC and AD subjects (panels a and t).

### NNMT Is Expressed in Cholinergic Neurones of the Hippocampus

**Fig. 3:**
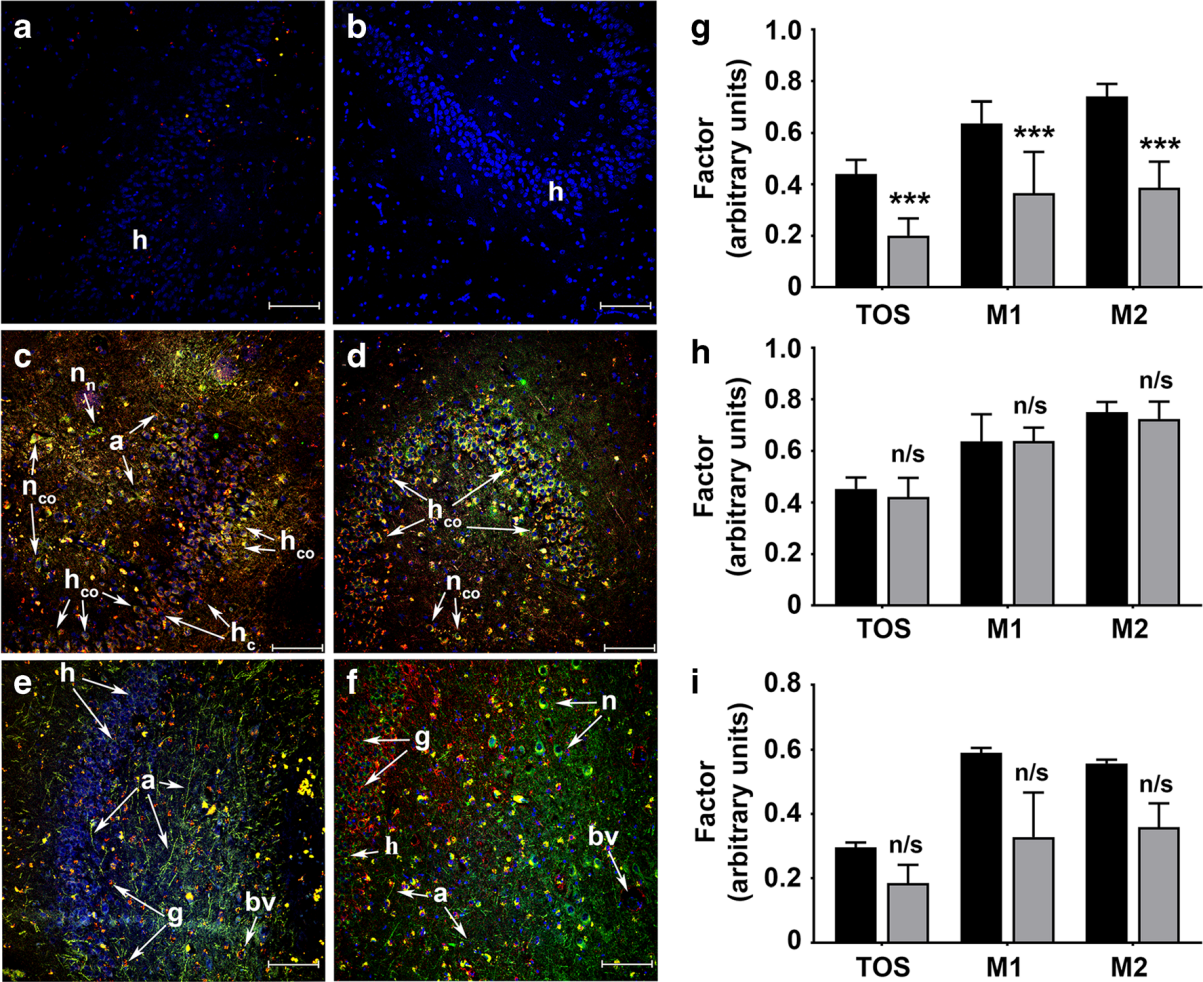
NNMT is expressed within cholinergic neurones in the hippocampus. **a**–**g** Representative maximum projection images of hippocampus from NDC and AD subjects. The expression of NNMT, the cholinergic neurone marker ChAT and the glial marker GFAP were detected using antibodies against each and visualised using AlexaFluor-labelled secondary antibodies and visualised using dual-label confocal microscopy. Counterstaining was performed using DAPI. NNMT expression is shown as green fluorescence, cell marker expression as red fluorescence and colocalisation as yellow fluorescence. Cell nuclei are shown as blue fluorescence. **a** and **b** Staining in the absence of primary antibodies. **c** and **d** NNMT and ChAT staining in NDC and AD subjects, respectively. **e** and **f** NNMT and GFAP staining in NDC and AD subjects, respectively. For all panels, h, hippocampal neurones; n_n_, neurone expressing NNMT; n_co_, neurone showing colocalisation between NNMT and ChAT; h_co_, hippocampal neurone showing colocalisation between NNMT and ChAT; h_c_, hippocampal neurone expressing ChAT; g, glial cell; a, axon; bv, blood vessel. For each, scale bar = 100 μm. **g**–**i** Quantification of NNMT colocalisation with ChAT and GFAP. Colocalisation coefficients TOS, M1 and M2 were calculated using the ImageJ plugin EzColocalization and expressed as ± SD. Statistical comparisons comprised multiple *t* tests using Holm-Sidak corrections for multiple comparisons. **g** Comparison of ChAT (black bars, *n* = 8) and GFAP (grey bars, *n* = 7) in all subjects. **h** Comparison of ChAT colocalisation coefficients in NDC (black bars, *n* = 5) and AD (grey bars, *n* = 3) subjects, respectively. **i** Comparison of GFAP colocalisation coefficients in NDC (black bars, *n* = 3) and AD (grey bars, *n* = 3) subjects, respectively. For all panels, n/s, not significant; ***P < 0.001

Having shown that NNMT expression was solely neuronal in the MTL and that these neurones may be of cholinergic phenotype, the next step was to confirm this in the hippocampus using dual-label confocal microscopy (Fig. 3a–f). Omission of primary antibodies revealed fluorescence due to DAPI counterstain only (panels a and b). NNMT expression was solely neuronal and was present within the cell body and axonal processes in both NDC and AD subjects (panels c–f). NNMT co-localised with ChAT expression in granule and molecular layer neurones of the hippocampus in NDC subjects; however, some neurones in both layers expressed either NNMT or ChAT only (panel c). In AD subjects, NNMT and ChAT were co-localised in all granule and molecular layer neurones (panel d). NNMT was not expressed in glial cells as evidenced by its lack of colocalisation with GFAP in either NDC or AD subjects (panels e and f). In NDC subjects, GFAP staining was sparse, with cells presenting a rounded morphology (panel e). In contrast, AD subjects presented significant gliosis, with extensive GFAP staining surrounding the neurones of the granule and molecular layers (panel f).

Our next step was to quantify colocalisation by calculating TOS and Manders’ colocalisation coefficients M1 and M2 (Fig. 3g). TOS for ChAT and NNMT colocalisation revealed moderate correlation (0.44 ± 0.054), most likely arising from NNMT expression throughout the cell body and axons whilst ChAT expression was predominantly within the cell body with little axonal expression (panels d–f). M1 and M2 values revealed that the majority of NNMT co-localised with ChAT with the converse also being true (63.7 ± 8.4% and 74 ± 4.9%, respectively). In contrast, TOS for GFAP and NNMT was low (0.2 ± 0.067), suggesting little or no colocalisation between the two proteins. Likewise, only a small amount of NNMT localised with GFAP, with the converse also true (36.6 ± 16% vs. 38.7 ± 10.2%). Statistical analysis showed that TOS, M1 and M2 values for GFAP were significantly lower than those for ChAT (*P* < 0.001 for all three values, *n* = 8 for ChAT and *n* = 7 for GFAP).

Our final step was to determine whether there were any differences in NNMT colocalisation with ChAT and GFAP between NDC and AD subjects (Fig. 3h–i). For ChAT, there was no change in colocalisation with NNMT expression, with TOS (0.45 ± 0.044 vs. 0.42 ± 0.073, *P* = 0.86), M1 (63.7 ± 10.5% vs. 63.8 ± 5.2%, *P* = 0.99) and M2 (75 ± 4% vs. 72.4 ± 6.7%, *P* = 0.86) not significantly different in NDC and AD subjects (*n* = 5 and 3, respectively). Likewise, for GFAP, there was no change in colocalisation with NNMT expression, with TOS (0.3 ± 0.015 vs. 0.19 ± 0.056, *P* = 0.06), M1 (59 ± 1.5% vs. 32.8 ± 13.9%, *P* = 0.06) and M2 (55.7 ± 1.2% vs. 35.9 ± 7.4%, *P* = 0.03) not significantly different in NDC and AD subjects (*n* = 3 for both).

## Discussion

This small-cohort proof-of-principle study is the first to demonstrate the increased expression of NNMT in specific areas of the brain in AD. The only other neurodegenerative disease in which NNMT expression has been extensively investigated is PD, in which NNMT expression was elevated in the granular layer of the cerebellum and in the caudate nucleus [8, 9]. In these two earlier studies, we demonstrated expression of NNMT mRNA and protein and assayed the activity of the enzyme in human NDC temporal lobe, which was the third highest after the spinal cord and the medulla [8]. We also reported that NNMT expression was solely neuronal, with extensive staining within the cell body and axons [8]. This present study extends this by showing that NNMT expression is increased in AD MTL. Also, this increase is present only in the area of the brain investigated which demonstrates disease pathology as evidenced by the lack of increase in expression of NNMT in the cerebellum, a brain region whose involvement in AD pathology is secondary to that observed in the cerebral cortex [25]. The increased expression of NNMT protein in AD patients, and its solely neuronal expression in both groups, mirrors what we have previously reported in PD brain [8, 9].

The dysregulation of NNMT expression has been described in several diseases. Elevated NNMT expression is a feature of numerous cancers [16, 17] in which it plays a central role in inducing cancer metastasis [26–28] along with changes in cell biochemistry and cellular signalling which are of significant survival benefit to the tumour cell [10–12, 29–31]. An increase in NNMT expression has also been linked to the pathogenesis of obesity [32, 33], with small molecule NNMT inhibitors shown to have efficacy at reversing the disease in animal models of obesity [34, 35].

Reduced NNMT expression has been associated with two neurological diseases. The first is bipolar disorder, in which serum NNMT levels were significantly lower in bipolar patients compared to non-psychiatric controls, an effect which was ascribed to the regulation of lipid metabolism by NNMT [36]. The second neurological disorder, schizophrenia, is associated with the single nucleotide polymorphism (SNP) rs694539, which is situated in the 5′ region of *NNMT* some 5 kb upstream of the transcription start site of the gene [37]. NNMT mRNA levels in post-mortem frontal cortex, reported in the same study, revealed a 33% decrease in expression. Although this study did not examine NNMT protein or activity levels, the authors suggested that NNMT is involved in the aetiology of schizophrenia via its regulation of cellular homocysteine concentrations, which are increased in the plasma of schizophrenia patients [38]. Further studies have confirmed the link between rs694539 and increased schizophrenia risk in women [39].

The most recent major meta-analysis of the relationship between serum homocysteine concentration and the risk of developing AD clearly showed a positive association between the two [40]. Souto and colleagues, using a genome-wide linkage analysis study, reported a significant association between rs694539 and elevated homocysteine concentration, which they suggested may be of relevance to the development of AD [41]. They proposed that increased expression of NNMT resulted in the increased consumption of methyl groups in the formation of MeN, which otherwise would have been available for the metabolism of homocysteine. Inhibitors of NNMT have been shown to decrease homocysteine levels in white adipose tissues of mice, with the converse occurring when treated with the NNMT inducer nicotinamide [42]. Nevertheless, there is, as yet, no evidence that either base in rs694539 influences NNMT expression. The recent study of Roostaei and colleagues has subsequently cast doubt upon a causal association between genetically determined homocysteine levels and the risk, severity and progression of AD [43], although non-genetic determinants of elevated homocysteine levels may still confer AD risk [44].

To date, the only study which has investigated a direct link between NNMT and AD is that of Olah and colleagues, who reported a direct interaction between NNMT and oligomeric Aβ [45]. This interaction was identified in cell-free extracts incubated with immunomobilised Aβ using protein microarrays. Although this demonstrated the potential for an interaction between the two proteins, such interaction was not investigated in whole cells or tissues and thus may not occur in situ. Hence, our study is the first to directly demonstrate a dysregulation of NNMT expression in AD patient brain.

It remains unclear whether the overexpression that we observe contributes towards, or is a stress response of the neurone to, the AD pathogenic process. Studies of the NNMT homologue ANMT-1 in *Caenorhabditis elegans* reveal mixed effects upon neuronal survival and organism lifespan which are dependent upon organism age. Overexpression of ANMT-1 increased autophagy during ageing, which maintained neuronal function in aged animals [46]. Overexpression of ANMT-1 also extended lifespan by increasing the production of MeN, the substrate for the aldehyde oxidase homologue GAD-3, which promotes longevity via the generation of the mitohormetic signal hydrogen peroxide [47]. Likewise, downregulation of ANMT-1 inhibited longevity extension mediated by the sirtuin-1 homologue sir-2.1 [47], an effect arising from the increased concentration of the sirtuin inhibitor nicotinamide [12]. However, in younger animals, high ANMT-1 activity induced abnormal behaviour by disrupting neuronal homeostasis and dopamine signalling [46]. ANMT-1 has only a 25.5% amino acid identity and 44.9% amino acid similarity with human NNMT, plus it has an additional 8 amino acids. Hence, it is unclear as to whether such effects are replicated in human cells. A number of studies have shown that NNMT expression is increased as a survival response to disease pathogenesis in diverse diseases including inflammation, cirrhosis, hepatitis, thrombosis and chronic obstructive pulmonary disease [48–52]. In our studies using cellular models, we demonstrated that overexpression of NNMT resulted in several cytotrophic effects such as an increase in complex I activity and ATP production [10] and an increase in both the number of neurite branches and functional synapses [13]. These effects were mediated via activation of the Akt/ephrin B2 signalling pathway [13] and the induction and activation of sirtuin-3 [12]. These findings have contributed to the changed perception of the role of NNMT from being solely a contributor to phase II metabolism to one of playing an important role in fundamental processes essential to cell survival.

The significant increase in NNMT in the MTL only, and not in the cerebellum, of AD patients points towards NNMT expression increasing in response to the AD disease process; otherwise, one would expect NNMT to be significantly higher in AD cerebellum also. This increased expression in the MTL was associated with Braak stage VI subjects only, i.e. AD patients. However, due to the small number of subjects in this study, there was only a small number of subjects in Braak stages 0 and I and none in Braak stages III, IV and V. Likewise, there were only a small number of cases for which CERAD score and neuropathological comorbidity data were available, although where such information was available increased NNMT expression was, once again, associated with high severity scores in these two measures. Hence, based upon this cohort, it was not possible to determine conclusively whether the increase in NNMT expression observed was associated solely with late-stage disease or whether expression increased as the severity of AD pathology increased. Additionally, no study has investigated the changes in NNMT expression in mild cognitive impairment, a precursor to the development of AD [53] which is associated with early Braak stage pathology [54]. Therefore, care must be taken in translating these findings to the role of NNMT in AD disease initiation and progression.

The lower expression of NNMT in the MTL compared to the cerebellum of NDC patients raises the possibility that regions associated with low NNMT expression may be more vulnerable to neurodegeneration. To our knowledge, no investigation of NNMT expression globally throughout the brain has been conducted, although we have previously investigated the expression of NNMT protein in the caudate nucleus, a region of the brain which demonstrates PD pathology [55]. We showed that NNMT expression in the caudate nucleus of NDC patients was higher than that observed in the cerebellum [8]. Hence, the evidence to date is insufficient to determine whether low levels of NNMT expression result in susceptibility to neurodegeneration.

Hence, it is conceivable that increased NNMT expression is an attempt to maintain synapse formation and function by increasing ATP supply and activating cell signalling pathways which lead to increased synapse formation. It is also possible that increased NNMT expression is in response to changes in cellular biochemistry such as increased oxidative stress, which is present in AD neurones [56]. We have recently shown that NNMT expression in SH-SY5Y human neuroblastoma cells decreased oxidative stress [29]. Another possibility is that of abnormal mitochondrial energy metabolism. Studies have shown that PC-12 cells exposed to Aβ demonstrate reduced ATP generation [57], mediated via its interaction with the mitochondrial membrane [58]. Furthermore, deficiencies in complex IV of the electron transport chain have been shown in vitro, in vivo and in post-mortem human brain to be an inherent feature of AD [59–62]. Our in vitro studies have shown that increased NNMT expression increased ATP synthesis [10–12]. Although we did not investigate its effect upon complex IV activity, we did show that NNMT increased ATP synthesis alongside an increase in the activity of complex I of the electron transport chain [10, 12].

Another possible effect of increased NNMT expression is the activation of the Akt signalling pathway. Akt is a cell survival kinase whose dysregulation is reported in neurodegenerative diseases such as PD and AD [63, 64]. Akt phosphorylation of downstream targets regulates a variety of cellular processes, one of which is glycogen synthase kinase-3β (GSK-3β), which phosphorylates tau [65]. The Akt pathway is downregulated in AD [66], leading to the overactivity of GSK-3β which, in turn, results in the hyperphosphorylation of tau [67]. Our in vitro studies have shown that NNMT activates the Akt pathway via ephrin-B2 [13]. Hence, it is possible that the increase in NNMT expression that we observed in these AD patients is an attempt of the neurone to protect against deleterious cellular and cytotoxic challenges. It also appears from our study that this is irrespective of the presence of neuropathological comorbidities.

The cohort in this study is small, comprising 19 subjects, although the age range of the groups was similar as was the distribution of the sexes of the subjects, which also mirror the prevalence of AD in the population. We have shown in previous investigations that the increase in NNMT we observed in a small PD cohort study (25 comprising 16 NDC and 9 PD) was replicated in a subsequent larger study (91 comprising 38 NDC and 53 PD) [8, 9]. In addition, we used maximum projection images to quantify our colocalisation data, which is common for similar studies in the literature [18, 19, 68]. The use of maximum projection images, rather than z-stacks, for the quantification of NNMT colocalisation with ChAT and GFAP results in the loss of 3-dimensional structural localisation data which can lead to colocalisation artefacts [69]. For example, in Fig. 3f, it is clear that NNMT and GFAP do not colocalise significantly; however, the overlapping of cells expressing either NNMT or GFAP can potentially appear as colocalisation in the maximum projection image and may explain the degree of apparent colocalisation quantified for NNMT and GFAP. It is clear from Fig. 3 that the subcellular expression of NNMT, ChAT and GFAP within cells and their interrelationship is complex; however, the aim of this study was not to investigate this in detail but merely to demonstrate the cell-type expression of NNMT in the hippocampus. Hence, the use of maximum projection images for this study was suitable for such analysis.

Hence, if our results are replicated in a larger, accurately age-matched study, it raises the possibility of using NNMT-targeted therapies to treat AD. If elevated NNMT expression contributes to disease pathogenesis, inhibitors of NNMT may be effective in mitigating this. NNMT is currently the focus of intense drug discovery studies for developing NNMT inhibitors as anti-cancer therapeutics [30, 70–72]. Conversely, if elevated NNMT expression is a stress response of the neurone to disease pathogenesis, therapies which induce, or mimic, elevated NNMT expression may be of benefit. One approach may be the regulation of NNMT expression via the targeting of the transcription factor Stat3. Stat3 activity is induced as a response to oxidative stress, reducing autophagy and mitophagy [73, 74]. We have shown that inhibiting Stat3 dimerisation reduced NNMT expression [75]; hence, activators of Stat3 may have the opposite effect. Nevertheless, this approach may not be desirable, because the activation of a common transcription factor such as Stat3 may lead to significant off-target effects. Other approaches may include gene delivery of the *NNMT* gene, or pharmacologically mimicking NNMT expression and/or activity. Recently, Fu and colleagues have shown that intragastric injection of MeN for 3 weeks ameliorated Aβ_1–42_-induced cognitive deficits in mice via inhibition of inflammation and apoptosis mediated by NF-κB signalling [76]. If NNMT expression could be increased during the prodromal phase of AD, it is possible that neurone integrity and function would be rescued and/or maintained before symptomatic onset. Advances in biomarker research may eventually allow for the identification of patients suitable for such interventions.

## Conclusions

Our study is the first to show that NNMT expression is elevated in the AD patient brain and that it is expressed in ChAT-positive neurones of the hippocampus. This increase in expression may contribute to disease pathogenesis, possibly via the regulation of cellular homocysteine levels, or it may be a stress response of neurones to changes in synaptic function and cellular biochemistry such as oxidative stress and a reduction in ATP supply. These results, if replicated in a larger cohort study, raise the possibility that NNMT is a viable therapeutic target for treating AD. Although such therapies will not cure the disease, they will slow down, or even stop, the disease pathogenic process, which would contrast with current therapies which solely address the symptoms of AD.

## Supplementary Information

ESM 1 (DOCX 54 kb)

## Data Availability

The datasets used and analysed during the current study are available from the corresponding author on reasonable request.

## Abbreviations

AD: Alzheimer’s disease
Aβ: Amyloid-β
ChAT: Choline acetyltransferase
DAB: 3,3′-Diaminobenzidine
GFAP: Glial acid fibrillary protein
GSK-3β: Glycogen synthase kinase-3β
MeN: 1-Methylnicotinamide
MTL: Medial temporal lobe
NDC: Non-disease control
NNMT: Nicotinamide N-methyltransferase
PD: Parkinson’s disease
TOS: Threshold overlap score
