# Supplementary material for: High Expression of Nicotinamide N-Methyltransferase in Patients with Sporadic Alzheimer’s Disease
Source: Mol Neurobiol. 2021 Jan 2;58(4):1769–81. doi: 10.1007/s12035-020-02259-9 (PMC7932959; doi:10.1007/s12035-020-02259-9)
Supplement: Supplementary file 1 — (DOCX 54 kb) [file 12035_2020_2259_MOESM1_ESM.docx]

|  | Male | n | Female | n | P-value |
| --- | --- | --- | --- | --- | --- |
| **Whole cohort** | | | | | |
| MTL | 3.8 ± 2.1^a^ | 7 | 2.3 ± 1.0 | 12 | 0.53 |
| CBM | 5.8 ± 2.0 | 7 | 2.8 ± 1.3 | 12 | 0.22 |
| **AD** | | | | | |
| MTL | 6.4 ± 3.1 | 4 | 3.8 ± 1.7 | 6 | 0.51 |
| CBM | 3.0 ± 2.1 | 4 | 4.3 ± 2.4 | 6 | 0.71 |
| **NDC** | | | | | |
| MTL | 0.37 ± 0.09 | 3 | 0.79 ± 0.32 | 6 | 0.26 |
| CBM | 9.6 ± 2.3 | 3 | 1.3 ­± 0.36 | 6 | 0.069 |

**Table S1. Comparison of NNMT expression between male and female subjects.** Whole cohort refers to all subjects, AD and NDC refers to stratification of cohort in accordance with disease status. ^a^NNMT expression is expressed as NNMT:tubulin ratio ± SEM. MTL = medial temporal lobe; CBM = cerebellum; AD = Alzheimer’s disease subjects; NDC = non-disease control subjects.

|  | Slope^a^ | R^2^ | n | P-value |
| --- | --- | --- | --- | --- |
| **Whole cohort** | -0.39 ± 0.27 | 0.12 | 18 | 0.16 |
| **AD** | -0.44 ± 0.33 | 0.19 | 10 | 0.21 |
| **NDC** | -1.78 ± 2.68 | 0.07 | 8 | 0.53 |

**Table S2. Correlation between NNMT expression in medial temporal lobe and cerebellum.** ^a^Expressed as ± SEM.

|  | Slope^a^ | R^2^ | n | *P* value |
| --- | --- | --- | --- | --- |
| **Whole cohort** | | | | |
| MTL | -0.06 ± 0.09 | 0.03 | 19 | 0.5 |
| CBM | -0.08 ± 0.1 | 0.03 | 19 | 0.58 |
| **AD** | | | | |
| MTL | 0.18 ± 0.2 | 0.1 | 10 | 0.38 |
| CBM | -0.41 ± 0.16 | 0.44 | 10 | 0.036 |
| **NDC** | | | | |
| MTL | -0.05 ± 0.01 | 0.8 | 9 | 0.001 |
| CBM | 0.07 ± 0.15 | 0.03 | 9 | 0.67 |

**Table S3. Correlation between NNMT expression and age at death.** ^a^Expressed as ± SEM.

|  | Slope^a^ | R^2^ | n | *P* value |
| --- | --- | --- | --- | --- |
| Whole cohort | 0.02 ± 0.07 | 0.008 | 13 | 0.77 |
| NDC | -0.01 ± 0.003 | 0.47 | 5 | 0.2 |
| AD | 0.01 ± 0.14 | 0.0001 | 8 | 0.95 |

**Table S4. Correlation between NNMT expression in the medial temporal lobe and *post mortem* interval.** ^a^Expressed as ± SEM.
